# Preparing Children for Hearing Examination in a Playful Way—Co-Creation and Evaluation of an App

**DOI:** 10.3390/jcm15020552

**Published:** 2026-01-09

**Authors:** Signe Wischmann, Lone Jantzen, Nete Rudbeck Kamper, Daniel Boonma Reipur, Margit Kabza, Maiken Bonne Jørgensen, Stefania Serafin, Per Cayé-Thomasen, Lone Percy-Smith

**Affiliations:** 1 Ear, Nose and Throat (ENT) and Audiology Clinic, Copenhagen Hearing and Balance Center, Rigshospitalet, Inge Lehmanns Vej 8, 2100 København, Denmark; lone.jantzen@regionh.dk (L.J.); nete.rudbeck.kamper@regionh.dk (N.R.K.); margit.kabza@regionh.dk (M.K.); stefania.serafin@regionh.dk (S.S.); per.caye-thomasen.01@regionh.dk (P.C.-T.); lone.percy-smith@regionh.dk (L.P.-S.); 2 Multisensory Experience Lab, Aalborg University Copenhagen, A.C. Meyers Vænge 15, 2450 København, Denmark; daniitinnakorn@gmail.com; 3 Independent Researcher, 2610 Rødovre, Denmark; 4 Independent Researcher, 2650 Hvidovre, Denmark; maikenbonne@icloud.com; 5 Department of Nordic Studies and Linguistics (NorS), University of Copenhagen, Njalsgade 136 & Emil Holms Kanal 2, 2300 København, Denmark

**Keywords:** hearing examination, children, preparation, hospital, app

## Abstract

**Background/Objectives**: Providing children with information about their treatment can help reduce uncertainty and anxiety associated with hospital procedures. The aim of this study was to develop and evaluate an app designed to prepare young children and their parents for a hearing examination in a playful and engaging way. **Methods**: This exploratory study adopted a participatory design approach. Children, parents, and clinicians co-created the app, and evaluations were conducted through focus group meetings, dialogue meetings, and surveys. **Results**: Children, parents, and clinicians evaluated the app positively. Findings indicated that children who used the app before their hearing examination met audiologists’ expectations to a greater extent than those who did not. However, no significant differences were found between the two groups regarding satisfaction with the examination, the children’s sense of safety, or parents’ prior knowledge of the procedure. The study also revealed implementation challenges: only 20% of children visiting the department had used the app beforehand, and funding for ongoing maintenance was not adequately addressed. **Conclusions**: Preparing children for hearing examinations with an app appears promising. However, to ensure the quality, accessibility, and sustainability of digital healthcare solutions, challenges related to implementation and maintenance must be considered in future studies.

## 1. Introduction

Pain, uncertainty, and disruption to normalcy can make meetings with hospitals a challenging and stressful experience for children and their parents [1,2,3]. Negative experiences are likely to affect the child’s coping with future treatments [4], leading to fear of healthcare professionals, unsuccessful follow-up appointments, and delayed recovery [3,5].

Providing children with information about their treatment can reduce uncertainty and anxiety regarding hospital procedures [5,6,7,8], while also improving parental satisfaction with the overall experience [9,10]. It is important, that the information is provided in a language and format the child can understand [2,5]. A study examining the assessment of preparatory procedural information showed that only 30% of the children felt they had adequate information about their upcoming procedure, even though 93% preferred to know as much about the procedure as possible [11]. Furthermore, only 35% of the parents in the study felt they knew enough about their child’s upcoming procedure [11].

Over the past decades, the approach to pediatric healthcare has developed. Awareness and emphasis are increasingly placed on children’s rights and competences, and initiatives are being implemented to improve children’s experiences at hospitals [4]. In 2017, the World Health Organization (WHO) published seven checklists to help hospitals assess child rights standards [12] in line with the Convention on the Rights of the Child [13]. The standards include the right to information, the right to be heard and involved in decisions about their treatment, and the right to play and leisure as part of the care environment [12].

Providing information about procedures and interventions is a prerequisite for involving children in decisions. Information should be engaging and easily accessible [5]. Digital apps built on game-based approaches can therefore be a relevant and useful media to provide information to both children and their parents [14]. Communication with public authorities is increasingly based on digital platforms [15], and in countries like Denmark, 93% of citizens aged 16 to 74 read information from authorities, including the healthcare sector, on a screen [16]. It is important though, that the quality, accessibility, and affordability of the digital solutions are well-tested [15]. Recent studies investigating the use of apps to prepare children for various medical procedures have reported positive results [4,7,17,18,19]. In a study by Bray et al. (2024), a preparatory information app was developed for children going to a blood test [18]. The authors found that the app improved children’s experience of the procedure, informed parents what would happen at the blood test, and enhanced parents’ ability to support their child [18]. Williams and Greene (2015) investigated an app addressing anticipatory anxiety in children aged 4–8 years going to medical imaging procedures and found that it both reduced compliance issues, the time preparing the child for imaging, and the anxiety level of the child [7]. In another study, focusing on preparation for MRI, Runge et al. (2018; 2024) investigated a multi-faceted concept combining a preparatory app, trained pediatric radiographers, a children’s lounge where children could play with a toy-scanner, and a child-friendly environment in the MRI room [4,17]. Results showed that the child-centred care led to a reduction in the use of general anesthesia from 57% to 5% in children aged 4–6 years [4,17]. Looking at 20 years of research in play at hospitals, Gjærde et al. (2021) divided play interventions into four clinical categories: (1) procedures and diagnostic tests, (2) patient education, (3) treatment and recovery, and (4) adaptation [20]. They found that studies across all categories reported positive effects on pain, stress, and anxiety [20].

In Denmark, children’s hospital experiences are increasingly recognized as an important indicator of healthcare quality. This is particularly reflected in large investments in new departments and hospital buildings designed especially for children and their families [21]. Other initiatives supporting children’s right to information, decision-making, and play are also emerging. In 2023, the rehabilitation method Auditory Verbal Therapy (AVT) was implemented nationally as a government funded offer to all children with hearing loss [22]. The central part of AVT is playful learning, and trained AVT-specialists use play in guiding parents to support their child’s listening and spoken language development [22]. Since children with hearing loss require life-long monitoring and treatment, audiological departments have a responsibility to offer playful testing, training, and treatment to create positive hospital experiences. In line with this, an audiology department in Denmark has explored the use of game-based apps to provide information about hearing loss to adolescents and parents. Evaluations are positive, showing that both virtual reality and tablet-based apps can be effective in creating positive awareness and knowledge of hearing loss [14,23]. The purpose of the current project is to broaden and optimize the child-friendly approach at the department with a specific focus on preparing the youngest patients for hearing examination with a game-based app.

### 1.1. The Importance of Preparing for Hearing Examinations

Early identification of hearing loss is required for early technical and educational intervention [24]. The audiologists and audiology assistants performing hearing examinations rely on a battery of tests to obtain definitive results about the type, degree, and configuration of the child’s hearing loss to be able to fit the child with the right technological solution [25]. The test procedure is challenging as it depends on continued interest and cooperation of the child [26]. Accordingly, the goal for audiologists and parents is to make the examinations as quick, recognizable, playful, and motivating for the child as possible [25]. At the audiology department the audiologists have a lot of different pedagogical methods to make the youngest children feel safe and to keep their concentration for as long as possible, so reliable hearing thresholds can be established [25]. However, clinical experience shows that children can still feel anxious or overwhelmed when they come to the hearing examination, especially if they have previously had negative hospital experiences, or if they have other diagnoses, i.e., autism spectrum disorders [27]. If the child cannot perform the hearing examination, it must be rescheduled, and the optimal technical and educational intervention, which is crucial for the child’s auditory and spoken language development [22], is delayed.

### 1.2. Aim

The overall aim of the study was to create easily accessible and child-friendly information about hearing examinations to provide children and their parents with positive and realistic expectations of the procedure. The specific objectives were to develop an app to prepare children aged 2–5 years for hearing examinations, and to evaluate its relevance and clinical applicability. In this article the following research questions are addressed:

1. What are considered, by the users, as the important key elements and content of an app designed to prepare young children for hearing examinations?
2. How do children, their parents, and clinicians evaluate an app as preparation for hearing examinations?
3. What are the differences and similarities between children and parents who have prepared with an app compared to children and parents who have not?

## 2. Materials and Methods

The study was conducted as a collaborative research project between an audiology department at a Danish hospital and a university-based multisensory research lab. It was conducted in accordance with the Danish Code of Conduct for Research Integrity from the Ministry of Higher Education and Science, and it was registered in the research record of the Capital Region of Denmark (P-2023-135). The study was notified to the Committees on Health Research Ethics for the Capital Region of Denmark but did not require ethical approval (F-23008133). Participation in the study was voluntary and based on informed written consent. The study was explorative and had a participatory design approach with a specific focus on user involvement and co-creation. It was structured in two phases: an app-development phase and an app-evaluation phase.

### 2.1. App-Development

#### 2.1.1. Participants

A total of 53 subjects were involved in the study. Fifteen children, their parents, two experienced audiologists, and six clinical speech and language pathologists participated in the app-development. Children were between 1½ and 6 years of age, and they were evenly distributed across this age span. Six of the fifteen children had a diagnosed hearing loss and regularly visited the department for hearing examinations and treatment. Children with typical hearing were included in the development phase, as hearing examinations are routinely performed not only for children with hearing loss but also for those considered at risk or suspected of hearing loss, for example, siblings.

#### 2.1.2. Process

Three speech and language pathologists from the research unit and one developer were the primary responsible for the app-development. The first step was defining the content of the app. Interviews were conducted with two clinical audiologists with extensive knowledge of hearing examinations to learn from their experiences. Additionally, the project members did live observations and watched film recordings of hearing examinations at the department.

When the overall content was decided, a draft for a voice-over script was tested and refined with the audiologists. At this point in time other departments at the hospital were contacted to explore if there already existed a child-friendly mascot or a design guide, which could be used in the app. There was no general mascot, so the appearance of the mascot was decided by the project members. The colour scale was inspired by colours used at a new hospital building for children and families.

A prototype of the app was created and tested with two clinicians and two children. The prototype did not have any sound, so a project member verbally guided them through the app. The app was updated and elaborated based on feedback from the four test participants.

Next, five children with typical hearing were asked to listen to different voices and choose their preferred ones for the app. Based on their preferences, the app was updated with the selected voice recordings. Subsequently, a test period followed, where the app was continuously tested, elaborated, and updated. Testing was conducted with a total of 14 children, each with one parent, and eight clinicians. Testing was observed by project members in order to track the reactions of the test participants. All test participants were interviewed about the design, usability, and relevance of the app. The final app was called “Warble at hearing examination”.

**Figure 1:**
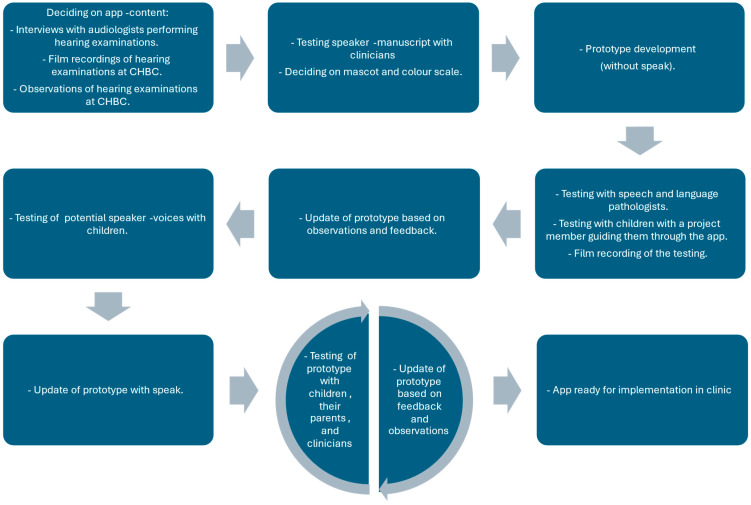
App-development process.

The development process is illustrated in Figure 1.

#### 2.1.3. Key Elements and Content of the App

The app was designed around three key elements, identified through the initial interviews with audiologists as particularly challenging or intimidating for young children undergoing hearing examinations: (1) the soundbox where the hearing examination is performed, (2) the white smocks that audiologists wear, and (3) clinicians touching the ears of the child. This final element was considered particularly important since otoscopy, objective measures, and the use of insert earphones to obtain ear-specific information of the hearing test, require clinicians to touch the ears of the child. The environment in the app was made as realistic as possible, with a slight cartoonish stylization (to avoid the uncanny valley), preparing children for the soundbox and audiologists in white smocks; the storyline resembles the real-life procedures with otoscopy, objective measurements, and hearing tests.

In the app the child meets the little mascot, Warble, who is going to a hearing examination. Warble is an animated imaginary animal inspired by a popular teddy bear (a Squishmallow) in neutral colours making it up to the imagination of the child to decide what it is. A digital audiologist guides Warble through the hearing examination and the child helps Warble perform otoscopy; remove earwax; measure emissions, middle ear pressure, and stapedius reflexes; and finally perform the hearing test. The most common hearing tests for children aged 2–5 years are Visual Reinforcement Audiometry (VRA) and Conditioned Play Audiometry (CPA) [28], so both tests are available for Warble to try. The game takes around 7 min to complete and ends with Warble receiving a small gift, reflecting the common practice of giving children a small reward after a hearing examination.

Warble is voiced by a 13-year-old girl, and a female audiologist from the audiology department is the voice of the digital audiologist. To make the speech authentic the audiologist naturally incorporated subtle phrases commonly used during hearing examinations.

**Figure 2:**
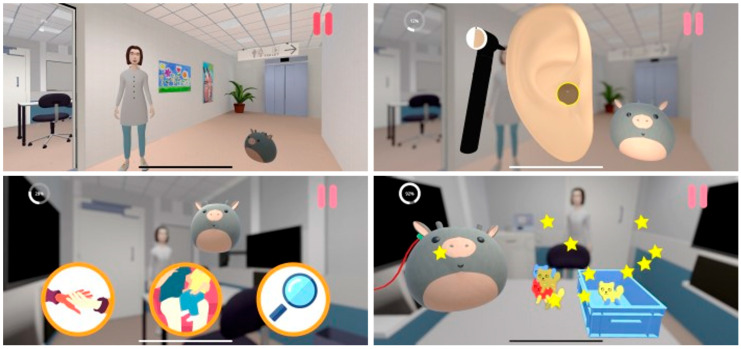
Screenshots from the “Warble at hearing examination” app.

Since the target group for the app is very young children, the design is kept simple, and the interactive touch areas are enlarged to make it easier for the children to tap, drag, or drop items. The app includes aspects of gamification and positive reinforcement like stars and encouraging pling sounds when successfully completing a task to motivate the child and create positive expectations of the hearing examination. The intention is that the child plays the game with a parent providing both with information about the hearing examination and creating a shared reference for talking about the procedure. Figure 2 presents screenshots from the app.

### 2.2. App-Evaluation

#### 2.2.1. Participants

Eight audiologists; a larger group of around thirty speech and language pathologists, nurses, medical doctors, researchers, and administrative staff from the department; and five speech and language pathologists from different municipalities participated in the evaluation of the app. Furthermore, all parents with children aged 2–5 years who came for a hearing examination at the audiology department during one week of October 2024 or during the two months March and April 2025, were invited to participate in the study. A total of 43 professionals, 6 children, and 49 parents participated in the evaluation of the study.

#### 2.2.2. Process

In addition to the evaluation, which was iteratively carried out during the co-creation process in the development phase, the app was further evaluated through focus group meetings, semi-structured interviews, and surveys.

As the primary stakeholders, audiologists were included in three focus group meetings throughout the project to evaluate the relevance and influence of the app, and to guide the implementation process. The first two meetings were conducted before the implementation of the app, and the last meeting took place after its implementation.

The secondary stakeholders included a larger group of clinicians (speech and language pathologists, nurses, and medical doctors), who participated in a monthly departmental meeting. This meeting also included other researchers and the administrative staff at the department. The app was presented by the project members, after which everyone downloaded the app and tried it out in small groups. When everybody had personal experience with the app, the meeting continued with feedback and an open dialogue about pros and cons of the app. All input and suggestions were noted by a project member. A similar procedure was used with stakeholders from the municipalities. Five speech and language pathologists with special knowledge of audiology participated in a dialogue meeting sharing their opinions of the app and suggestions for the implementation process.

Finally, the end users—children and their parents—were invited to share their thoughts of the app in semi-structured interviews and in a survey. The interviews were conducted during one week of October 2024 by an interviewer who was independent of the project, which reduced bias and encouraged participants to share their honest opinions. Parents of children aged 2 to 5 years, who visited the department for a hearing examination, were invited to participate in the interview. Participation required that they were acquainted with the app. Parents had either played “Warble at hearing examination” with their child at home, were willing to play it at the department before the interview, or had been introduced to the app prior to participation. The interview both included questions about the app and more general questions about children’s screen use and the use of digital solutions for providing healthcare information, as these factors potentially could influence whether the app would be used in practice. After the interviews, the interviewer analyzed and reported the findings to the research group.

The survey was part of a larger investigation of audiologists’, children’s, and parents’ general experience with hearing examinations, and it was developed and tested in close collaboration with the audiologists at the department. In March and April 2025, audiologists performing the hearing examination informed parents about the study and distributed questionnaires and consent forms. Children who were blind or who had parents that did not speak any Danish were excluded from the study. Parents, who agreed to participate, were asked to return the completed questionnaire and the signed consent form in a mailbox after the hearing examination. This procedure was chosen to avoid the risk of parents feeling obligated to participate, as saying no to a clinician might be uncomfortable [29]. Characteristics of the child and the hearing examination—including age, possible diagnosis, experience with hearing examinations, test duration, number of measured hearing thresholds, and objective measures performed—were completed by the audiologists. Furthermore, the audiologists assessed to which extent the hearing examination met their expectations. The evaluation was based on the audiologists’ prior knowledge of the child’s age, any existing diagnoses, and test experience, with expectations typically higher for older children without additional diagnoses. This subjective outcome measure was included as the number of hearing thresholds, or the test duration do not always reflect a successful hearing examination. For example, a longer test may indicate good cooperation, allowing the child to continue for an extended period; however, it might also reflect difficulties in obtaining sufficient thresholds. Similarly, achieving few thresholds using insert earphones or, for example, a combination of air- and bone-conduction thresholds can provide more clinically meaningful information than completing a full hearing test in free field.

In the questionnaire parents were asked if they knew what was going to happen at the hearing examination; how they experienced the hearing examination; how their child experienced the hearing examination; to what extent they believed that their child felt safe during the hearing examination; how their child was prepared for the hearing examination; if the child had played “Warble at hearing examination”; if they liked the game; and if they would recommend the game to others. The questionnaire was developed and validated for the project by two master thesis students.

Because the children were very young and likely to be tired after the hearing examination, the questionnaire for children included just one question “Do you like the game Warble at hearing examination?”. Inspired by a similar questionnaire developed and validated for children [4], a visual scale with emojis supplemented the answers “Yes, a lot”, “Yes, a little”, “No, not so much”, “No, not at all”, and “Don’t know”.

**Figure 3:**
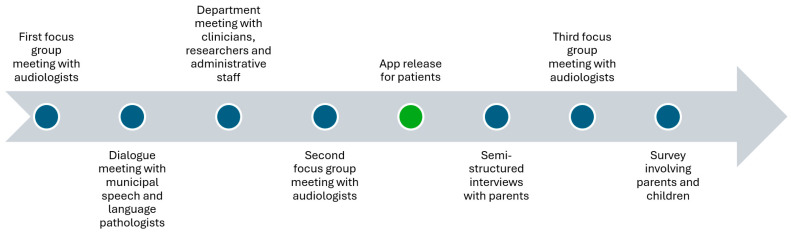
App-evaluation process.

The development process is illustrated in Figure 3.

### 2.3. Data Analysis

Due to the exploratory nature of the study and the small size of the Warble App group (*n* = 9), no formal inferential statistical tests were conducted to compare the Warble App group and the control group. Survey data are therefore reported descriptively using frequencies, percentages, means, and standard deviations. Group differences are presented for exploratory comparison only and should be interpreted as indicative rather than statistically conclusive. The qualitative findings from the co-creation process and focus group meetings are presented using descriptive summaries. All qualitative analysis and summaries were discussed among project members, led by the first author. Data from the semi-structured interviews and the survey are reported as frequencies and percentages.

## 3. Results

The oral evaluations of the app during focus group meetings and dialogue meetings were predominantly positive. The vast majority of both primary and secondary stakeholders evaluated the app as a highly relevant preparatory tool that facilitated a shared language among clinicians, children, and parents during the hearing examination. However, they also had a lot of good suggestions on how to improve the game with more advanced visuals, and additional new features and extra levels. Two clinicians shared case-stories about children who were previously considered untestable but after using the app completed the hearing assessment successfully. Moreover, there was a clear interest in identifying additional areas at the department where similar applications could be developed to support children’s preparation.

A total of five parents participated in the qualitative interviews. The children of the participating parents were also invited to take part in separate interviews, however none wished to participate. They were either too tired after the hearing examination or occupied in play. Only one parent had used the app with their child at home as preparation for the hearing examination, the others had no knowledge of it. Three of the remaining four parents played it with their child at the department right before the interview. All five parents understood the purpose of the app and appreciated that an app designed to prepare children for a hearing examination had been developed. All four parents who had tried the app prior to participation also experienced the usability as good but found a few errors in the software. All parents had general rules for screen use at home, and four of them reported that they often reflected on their child’s screen use. However, they distinguished between the purpose of the digital input and all five parents emphasized that they generally viewed digital solutions aimed at learning as positive. One parent suggested that the app should include a statement clarifying that it is non-commercial and does not collect personal information about the user, as this would reduce parents’ scepticism about downloading it. This was immediately incorporated in the short description of the app in the information material and in App Store and Google Play. Furthermore, the software was re-checked for errors.

A total of 89 parents were invited to participate in the survey in March and April 2025 and 44 parents accepted (49%). Of the 44 participants, 9 children (20%) had played Warble at hearing examination as preparation for the hearing examination. Children who had played Warble had a mean age of 56.9 months (SD = 14.9), and were therefore slightly older than the control group, whose mean age was 43.9 months (SD = 13.8). In the Warble group, 67 percent were girls compared to 41 percent in the control group. The audiologists assessed that all 9 children in the Warble group (100%) met their expectations for the hearing examination (answering either “all of what we expected” or “more than we expected”), while the number of children in the control group was 28 (65%). Characteristics of participants and their hearing examination are provided in Table 1.

Parents’ experiences of the hearing examination were overall positive with 78% (*n* = 7) of the Warble-group parents answering that the hearing examination was “very good” and 22% (*n* = 2) reporting “good”. Of the parents in the control group, 68 percent (*n* = 30) experienced the hearing examination as “very good”, 27 percent (*n* = 12) as “good”, 2 percent (*n* = 1) as “neither good nor bad”, and 2 percent (*n* = 1) as “bad”. Asking about their child’s experience of the hearing examination, 78% (*n* = 7) of the Warble group believed that their child had a “very good” experience and 22% (*n* = 2) believed it was “good”. The respective numbers for the control group were 55% (*n* = 24) “very good”, 32% (*n* = 14) “good”, and 14% (*n* = 6) “neither good nor bad”. The majority of parents in both groups experienced that their child felt safe during the hearing examination. In the Warble group 89% (*n* = 8) answered “to a high degree” and 11% (*n* = 1) answered “to some degree”, compared to 73% (*n* = 32) and 25% (*n* = 11) in the control group. One parent (2%) in the control group answered that their child felt safe “to a lesser degree”. Most parents knew what was going to happen at the hearing examination with 75% (*n* = 6) of the Warble group answering, “to a high degree” and 25% (*n* = 2) answering “to some degree”. For the control group, the numbers were 66% (*n* = 29) and 34% (*n* = 15), respectively.

**Figure 4:**
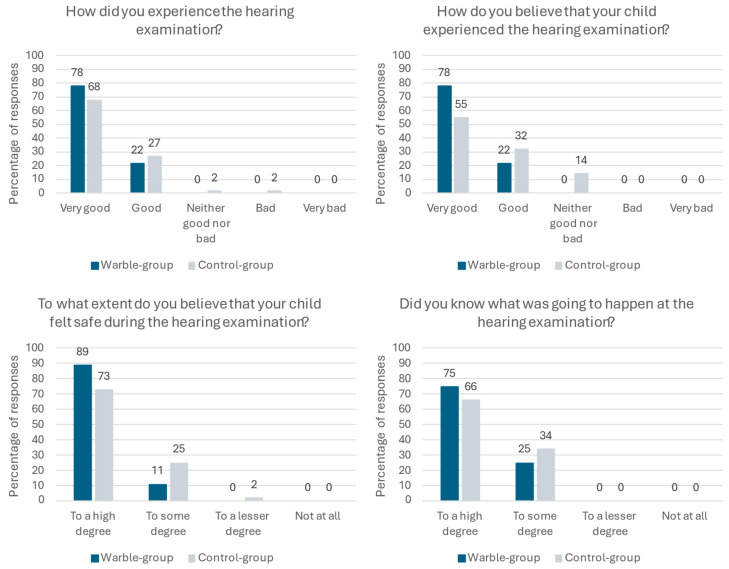
Parents’ and children’s experiences of hearing examinations. Although differences between groups are visually apparent in the figure, these differences were not subjected to formal statistical testing and should be interpreted descriptively.

Evaluating “Warble at hearing examination”, the parents were asked if they liked the game and if they would recommend it to others. A proportion of 63 percent (*n* = 5) liked the game “a lot” and 38 percent (*n* = 3) liked it “a little”. They would all (100%, *n* = 9) recommend the game for others with children going to a hearing examination. Asking the children the same question, 83% (*n* = 5) answered that they liked the game a lot and 17% (*n* = 1) liked it a little. Three children did not answer the questionnaire. The children had played the game 8.9 times (SD = 7.8) on average. Data from the survey are summarized in Table 2 and Figure 4.

## 4. Discussion

This study aimed at preparing children aged 2 to 5 years and their parents for a hearing examination in a playful way. The app “Warble at hearing examination” was co-created with clinicians, parents, and children, and its usability and relevance were evaluated. As reported in other studies we found that involving stakeholders in the development turned out to be positive [2,30]. Audiologists had the necessary expertise to identify key areas of the app, and the vast majority of stakeholders evaluated the final app as a child-friendly and a relevant way of preparing children for hearing examinations. Evaluating young children’s experiences and comfort can be challenging [2,4]. In this study we used systematic observations during the development phase to assess how children experienced the game. As previously reported by Williams and Greene (2015) [7], this procedure proved valuable in highlighting strengths and challenges, and in providing feedback and ideas for improving the game. In the evaluation phase questionnaires were included, and children were asked if they liked the game using a four-point Likert scale supported by emojis. All children and parents, who had prepared with “Warble at hearing examination”, stated that they liked the app, and all parents would recommend it for others. These findings are in line with other studies supporting the relevance of using apps to create easily accessible and child-friendly information about hospital procedures [4,7,17,18,19].

Providing parents with procedural information to share with their child is important, because parents have a key role in both facilitating and limiting their child’s knowledge of procedures [5,18]. However, our study highlighted challenges in implementing the app. Despite positive evaluations of the app, posters in the department, and a QR code in the invitation letter to the hospital, only 20% of parents and children going to the hearing examination had tried the app. This is less than reported in other studies [4] and calls for much more focus on the implementation phase. Bray et al. (2022) addresses the dilemma, that parents do not always access the information provided before their hospital visit even though both children and parents have a wish to be informed about planned procedures [11]. This project sought to involve all stakeholders from the beginning to the end of the project, based on the hypothesis that a co-creation design would reduce the gap from research to practice. Future studies may allocate equal focus to implementation, development, and evaluation to ensure preparatory apps reach children and parents.

In our study parents were asked to evaluate the hearing examination experience on behalf of their child to investigate if children, who had prepared with the app, experienced the hearing examination differently than children, who had not. However, descriptive comparisons did not indicate clear differences between the two groups regarding satisfaction with the examination, children’s perceived sense of safety, or parents’ prior knowledge of the procedure. Parents in both groups reported very high levels of satisfaction with the hearing examination, both for themselves and for their child. They all knew what would happen “to some” or “to a high degree”, and all but one parent stated that their child felt safe during the hearing examination. These positive results might reflect the overall focus on creating child-friendly and playful hospital experiences which have increased at the department over the past years. “Warble at hearing examination” is not an isolated initiative to reduce children’s uncertainty and anxiety about hospital procedures, but part of a more holistic approach to improve pediatric healthcare, which also includes child-friendly waiting areas, playful rehabilitation programmes, and pedagogical methods in assessment. In our study, we did, however, see a difference between the two groups in the audiologists’ expectations of the hearing examination. All children who had prepared with the app completed “all” or “more than expected” in the hearing examination compared to 65% of children who had not prepared with the app. However, as the Warble App group included only nine children, these numbers should be viewed as indicative.

In the interviews, parents viewed the development of an app to help them prepare their children for hearing examinations positively. They generally appreciated digital tools aimed at learning. Since most parents carry a smartphone, an app can be accessed at any time, making it easy for families to prepare for their appointment. However, an app may not be suitable for children under two years of age, as this age group might not yet have the motor skills required for drag-and-drop functions on a parent’s mobile phone. Future studies should explore ways to best prepare this age group for hearing examinations, as testing them in an audiometry booth often presents challenges.

A final finding of our study is the challenge of technical maintenance of an app, and it is of utmost importance to discuss this challenge. Updates on all hospital systems are typically carried out quarterly and this may disrupt an app run by the hospital’s technology department. Continuous monitoring of the app is important to avoid errors in the software, but monitoring is not for free. “Warble at hearing examination” was run as a non-commercial research project funded mainly for the development and evaluation phases and thus did not include a technology readiness level nine, i.e., the final implementation level in a clinical setting. Involving the technology department as stakeholders might ease implementation of apps developed in future research projects at hospitals. However, to ensure that the apps are ultimately used in clinical practice, funding for their implementation and maintenance should be planned from the early stages of the project. A challenge here is, however, that non-profit foundations typically do not cover maintenance expenses.

In Denmark as well as globally, there is a governmental push for digitalisation of health care information [15,16]. Therefore, our challenges provide useful data and important experience for policy makers in dealing with the implementation and maintenance challenges to avoid solid and well-tested digital solutions to end when funding ends. Future studies with this challenge in focus seem highly warranted.

### Limitations

Our study has several limitations. One important limitation is the limited sample size. Only nine children used the app prior to the hearing examination, which reduces the generalizability of the findings and prevented formal inferential statistical tests and subgroup analyses that could have clarified whether certain groups of children benefit more from an app than others. We know that parents and children who used the app were satisfied with it, but we did not follow up with the remaining parents to explore why they chose not to use the app. Potential reasons could include lack of time, a perception that the child did not need the app, or limited awareness of the app, as expressed by the parents in the semi-structured interviews. Future studies should place greater emphasis on the implementation phase to ensure that all families are aware of the app prior to the hearing examination, so that non-use reflects an active choice rather than lack of knowledge. Investigating reasons for non-use is essential to clarify the true need for preparational apps. Another limitation was the lack of funding for implementation and long-term maintenance of the app. Without a clear plan for sustainability, even successful apps risk remaining unused in clinical practice. To avoid wasting resources and missing opportunities to improve care, future research should integrate implementation strategies and secure funding models from the outset.

## 5. Conclusions

According to the Convention on the Rights of the Child, children have the right to information and to be involved in decisions about healthcare procedures and treatment. In this study we co-created and evaluated an app with the purpose of preparing young children and their parents for a hearing examination in a playful way. The app was well received by children, their parents, and relevant stakeholders, and children who had prepared using the app met audiologists’ expectations for the hearing examination to a greater extent than those who had not. However, the study revealed challenges related to implementation. Only 20% of children visiting the department used the app before the hearing examination, and funding for its ongoing maintenance was not properly addressed. To ensure the quality, accessibility, and affordability of digital health care solutions, these challenges must be addressed. Finally, it is worth noting that almost all children in our study felt safe during the hearing examination, whether they used the app or not. This clearly highlights the continued importance of prioritizing a holistic approach to creating child-friendly healthcare experiences.

## Figures and Tables

**Table 1:** Characteristics of participants and hearing examinations for the Warble App group and control group. The number and percentage of participants are provided for each group. The values presented are descriptive. No inferential statistical tests were applied to assess group differences due to the small and unequal sample sizes.

Characteristics	Warble Group, *n*	Control Group, *n*
Mean age (SD)	56.9 months (14.9)	43.9 months (13.8)
Sex		
Girls	6 (67%)	18 (41%)
Boys	3 (33%)	26 (59%)
Mean test duration (SD)	39 min (8.4)	45 min (11.1)
Experience with hearing examination		
First time	0 (0%)	3 (7%)
Second time	3 (33%)	0 (0%)
Third time	0 (0%)	9 (21%)
Fourth time	0 (0%)	6 (14%)
More than four times	6 (67%)	24 (57%)
Objective measures		
Cerumen removal	2 (22%)	10 (23%)
Otoscopy	8 (89%)	35 (81%)
Automated Auditory Brainstem Response (a-ABR)	0 (0%)	3 (7%)
Transient Evoked Otoacoustic Emissions (TEOAE), right ear	5 (56%)	27 (63%)
Transient Evoked Otoacoustic Emissions (TEOAE), left ear	5 (56%)	26 (60%)
Tympanometry/reflexes/leak, right ear	7 (78%)	39 (91%)
Tympanometry/reflexes/leak, left ear	8 (89%)	39 (91%)
Mean number of measured hearing thresholds (SD)	11.9 (5.5)	9 (4.8)
To which extent did the hearing examination meet expectation?		
Not at all	0 (0%)	0 (0%)
Less than half of the expected	0 (0%)	4 (9%)
Half of the expected	0 (0%)	2 (5%)
More than half of the expected	0 (0%)	9 (21%)
All of what we expected	5 (56%)	13 (30%)
More than we expected	4 (44%)	15 (35%)

**Table 2:** Parents’ and children’s experiences of hearing examinations in the Warble App group and control group.

Questions	Warble Group	Control Group
How did you experience the hearing examination?		
Very good	7 (78%)	30 (68%)
Good	2 (22%)	12 (27%)
Neither good nor bad	0 (0%)	1 (2%)
Bad	0 (0%)	1 (2%)
Very bad	0 (0%)	0 (0%)
How do you believe that your child experienced the hearing examination?		
Very good	7 (78%)	24 (55%)
Good	2 (22%)	14 (32%)
Neither good nor bad	0 (0%)	6 (14%)
Bad	0 (0%)	0 (0%)
Very bad	0 (0%)	0 (0%)
To what extent do you believe that your child felt safe during the hearing examination?		
To a high degree	8 (89%)	32 (73%)
To some degree	1 (11%)	11 (25%)
To a lesser degree	0 (0%)	1 (2%)
Not at all	0 (0%)	0 (0%)
Did you know what was going to happen at the hearing examination?		
To a high degree	6 (75%)	29 (66%)
To some degree	2 (25%)	15 (34%)
To a lesser degree	0 (0%)	0 (0%)
Not at all	0 (0%)	0 (0%)
How was your child prepared for the hearing examination?		
We talked about it at home	8 (89%)	32 (73%)
The child played with the “hearing-examination-installation” in the waiting area	9 (100%)	32 (73%)
We practiced with ear plugs at home	0 (0%)	2 (5%)
The child has been to a hearing examination before and knew what to expect	7 (78%)	32 (73%)
With the app “Warble at hearing examination”	9 (100%)	0 (0%)
The child did not know what was going to happen at the hearing examination	0 (0%)	6 (14%)
Did you like the game “Warble at hearing examination? (parents)		
Yes, a lot	5 (63%)	-
Yes, a little	3 (38%)	-
No, not so much	0 (0%)	-
No, not at all	0 (0%)	-
Would you recommend the game “Warble at hearing examination for others?		
Yes	9 (100%)	-
No	0 (0%)	-
I don’t know	0 (0%)	-
Did you like the game “Warble at hearing examination? (children)		
Yes, a lot	5 (33%)	-
Yes, a little	1 (17%)	-
No, not so much	0 (0%)	-
No, not at all	0 (0%)	-

## Data Availability

Data is unavailable due to privacy restrictions as participants have not given permission to share data with people not directly involved in this study.

## References

[1] Claridge A.M., Powell O.J. (2023). Children’s experiences of stress and coping during hospitalization: A mixed-methods examination. J. Child Health Care.

[2] Thestrup J., Sørensen J.L., Esbjørn B.H., Hybschmann J., Frandsen T.L., DeCosta P., Gjærde L.K. (2025). Paediatric patient perceptions of healthcare professionals: Contributions to a communication curriculum. Eur. J. Pediatr..

[3] Bray L., Sharpe A., Gichuru P., Fortune P.M., Blake L., Appleton V. (2020). The acceptability and impact of the Xploro digital therapeutic platform to inform and prepare children for planned procedures in a hospital: Before and after evaluation study. J. Med. Internet Res..

[4] Runge S.B., Precht H., Jensen I.E., Jensen K., Johannesen T.A., Pedersen M.R.V., Christensen N.L. (2024). Children Centered Care: Child and parent perspectives on a multi-faceted concept for magnetic resonance imaging without anesthesia—A survey. Pediatr. Radiol..

[5] Bray L., Appleton V., Sharpe A. (2019). ‘If I knew what was going to happen, it wouldn’t worry me so much’: Children’s, parents’ and health professionals’ perspectives on information for children undergoing a procedure. J. Child Health Care.

[6] Constantinescu-Sharpe G., Phillips R.L., Davis A., Dornan D., Hogan A. (2017). Social inclusion for children with hearing loss in listening and spoken Language early intervention: An exploratory study. BMC Pediatr..

[7] Williams G., Greene S. (2015). From analogue to apps-developing an app to prepare children for medical imaging procedures. J. Vis. Commun. Med..

[8] Orenius T., Säilä H., Mikola K., Ristolainen L. (2018). Fear of Injections and Needle Phobia Among Children and Adolescents: An Overview of Psychological, Behavioral, and Contextual Factors. SAGE Open Nurs..

[9] Wong C.L., Ip W.Y., Kwok B.M.C., Choi K.C., Ng B.K.W., Chan C.W.H. (2018). Effects of therapeutic play on children undergoing cast-removal procedures: A randomised controlled trial. BMJ Open.

[10] Delaney D., Bayley E.W., Olszewsky P., Gallagher J. (2015). Parental Satisfaction with Pediatric Preoperative Assessment and Education in a Presurgical Care Center. J. PeriAnesthesia Nurs..

[11] Bray L., Appleton V., Sharpe A. (2022). ‘We should have been told what would happen’: Children’s and parents’ procedural knowledge levels and information-seeking behaviours when coming to hospital for a planned procedure. J. Child Health Care.

[12] World Health Organization, Regional Office for Europe (2017). Children’s Rights in Hospital: Rapid-Assessment Checklists.

[13] United Nations General Assembly, Convention on the Rights of the Child, New York, 1989. https://www.unicef.org/child-rights-convention/convention-text.

[14] Wischmann S., Kamper N.R., Jantzen L., Hammer L., Reipur D.B., Serafin S., Percy-Smith L. (2024). Explaining neurological factors of hearing loss through digital technologies. Int. J. Pediatr. Otorhinolaryngol..

[15] World Health Organization (2019). WHO Guideline: Recommendations on Digital Interventions for Health System Strengthening, Geneva, 2019.

[16] Jacobsen A.V., Erenbjerg A., Törnfelt C., Tassy A. IT Usage Among the Population 2023, København, 2023. https://www.dst.dk/Site/Dst/Udgivelser/GetPubFile.aspx?id=49775&sid=itbef2023.

[17] Runge S.B., Christensen N.L., Jensen K., Jensen I.E. (2018). Children centered care: Minimizing the need for anesthesia with a multi-faceted concept for MRI in children aged 4–6. Eur. J. Radiol..

[18] Bray L., Ng S.M., Pyke L., Kikby J. (2024). Acceptability and feasibility of an app to prepare children for a blood test: An exploratory cohort study. Health Educ. J..

[19] Oulton K., Oldrieve N., Bayliss J., Jones V., Manning I., Shipway L., Gibson F. (2017). Using participatory and creative research methods to develop and pilot an informative game for preparing children for blood tests. Arts Health.

[20] Gjærde L.K., Hybschmann J., Dybdal D., Topperzer M.K., Schrøder M.A., Gibson J.L., Ramchandani P., Ginsberg E.I., Ottesen B., Frandsen T.L. (2021). Play interventions for paediatric patients in hospital: A scoping review. BMJ Open.

[21] Rigshospitalet (2016). Rigshospitalet: A World-Class New Hospital for Children, Adolescents, Expectant Mothers, and Their Families.

[22] Wischmann S., Samar C.F., Hestbæk M.K., Serafin S., Cayé-Thomasen P., Percy-Smith L. (2025). Quality assurance of a hospital-based auditory verbal intervention for children with hearing loss. J. Clin. Med..

[23] Wischmann S., Hammer L., Jantzen L., Sandager T.P., Kamper N.R., Henriksen S.T., Adjorlu A., Serafin S., Cayé-Thomasen P., Percy-Smith L. (2025). Virtual reality to create positive and timely awareness of hearing loss in middle- and secondary school. Int. J. Pediatr. Otorhinolaryngol..

[24] WHO (2021). World Report on Hearing, Geneva, 2021.

[25] Copenhagen Hearing and Balance Centre (2025). Assessments of Children.

[26] Psarommatis I., Valsamakis T., Raptaki M., Kontrogiani A., Douniadakis D. (2007). Audiologic evaluation of infants and preschoolers: A practical approach. Am. J. Otolaryngol. Head Neck Med. Surg..

[27] Beers A.N., McBoyle M., Kakande E., Santos R.C.D., Kozak F.K. (2014). Autism and peripheral hearing loss: A systematic review. Int. J. Pediatr. Otorhinolaryngol..

[28] American Academy of Audiology (2020). Clinical Guidance Document: Assessment of Hearing in Infants and Young Children, 2020.

[29] Larsen J., McMillin A. (2011). Ethical issues in the conduct of research at a multidisciplinary clinic. Semin Speech Lang..

[30] Vickers D., Salorio-Corbetto M., Driver S., Rocca C., Levtov Y., Sum K., Parmar B., Dritsakis G., Flores J.A., Jiang D. (2021). Involving children and teenagers with bilateral cochlear implants in the design of the BEARS (Both EARS) virtual reality training suite improves personalization. Front. Digit. Health.

